# Heritability of heart rate recovery and vagal rebound after exercise

**DOI:** 10.1007/s00421-016-3459-y

**Published:** 2016-09-10

**Authors:** Ineke Nederend, Nienke M. Schutte, Meike Bartels, Arend D. J. ten Harkel, Eco J. C. de Geus

**Affiliations:** 1 Department of Biological Psychology, Faculty of behavioral and Movement Sciences, VU University Amsterdam, Van der Boechorststraat 1, 1081 BT Amsterdam, The Netherlands; 2 EMGO + Institute for Health and Care Research, VU University Medical Center, Van der Boechorststraat 7, 1081 BT Amsterdam, The Netherlands; 3 Department of Pediatric Cardiology, LUMC University Medical Center, Albinusdreef 2, 2333 ZA Leiden, The Netherlands

**Keywords:** Heart rate recovery, Exercise, Heritability, Twin study

## Abstract

**Purpose:**

The prognostic power of heart rate recovery (HRR) after exercise has been well established but the exact origin of individual differences in HRR remains unclear. This study aims to estimate the heritability of HRR and vagal rebound after maximal exercise in adolescents. Furthermore, the role of voluntary regular exercise behavior (EB) in HRR and vagal rebound is tested.

**Methods:**

491 healthy adolescent twins and their siblings were recruited for maximal exercise testing, followed by a standardized cooldown with measurement of the electrocardiogram and respiratory frequency. Immediate and long-term HRR (HRR60 and HRR180) and vagal rebound (heart rate variability in the respiratory frequency range) were assessed 1 and 3 min after exercise. Multivariate twin modeling was used to estimate heritability of all measured variables and to compute the genetic contribution to their covariance.

**Results:**

Heritability of HRR60, HRR180 and immediate and long-term vagal rebound is 60 % (95 % CI: 48–67), 65 % (95 % CI: 54–73), 23 % (95 % CI: 11–35) and 3 % (95 % CI: 0–11), respectively. We find evidence for two separate genetic factors with one factor influencing overall cardiac vagal control, including resting heart rate and respiratory sinus arrhythmia, and a specific factor for cardiac vagal exercise recovery. EB was only modestly associated with resting heart rate (*r* = −0.27) and HRR (rHRR60 = 0.10; rHRR180 = 0.19) with very high genetic contribution to these associations (88–91 %).

**Conclusions:**

Individual differences in HRR and immediate vagal rebound can to a large extent be explained by genetic factors. These innate cardiac vagal exercise recovery factors partly reflect the effects of heritable differences in EB.

## Introduction

Low resting heart rate is associated with lower risk for cardiovascular and non-cardiovascular premature mortality (Greenland et al. 1999; Palatini and Julius 2004; Zhang and Zhang 2009). Mechanistic explanations for this protective effect often invoke a key role for the autonomic nervous system, in particular the parasympathetic branch. The resting heart rate is predominantly determined by cardiac vagal control, which can be measured noninvasively by heart rate variability (HRV) (1996; Berntson et al. 1993). High cardiac vagal control increases electrical stability of the heart (Bonilla et al. 2012; Schomer et al. 2014; Schwartz et al. 2003; Vanoli et al. 1991) and various HRV measures have been shown to be predictive of cardiac mortality and morbidity.

High cardiac vagal control further seems paramount in explaining the predictive effects of heart rate recovery (HRR) after exercise testing. HRR can be divided into immediate (1 min after exercise cessation) recovery and long-term recovery (>1 min after exercise cessation). During immediate recovery after maximal exercise, heart rate decreases mainly because of increased vagal control while sympathetic control remains practically unchanged (Imai et al. 1994; Lahiri et al. 2008; Ohuchi et al. 2000). High immediate HRR after exercise cessation is associated with lower cardiac mortality in various patient groups (Bigger, Jr. et al. 1993; Diller et al. 2006; Groarke et al. 2015; Kleiger et al. 1987; Minkkinen et al. 2014; Nissinen et al. 2003; Panaite et al. 2015; Watanabe et al. 2001) but also in healthy individuals (Cole et al. 1999, 2000; Jouven et al. 2005; Lovallo 2015; Mora et al. 2005; Tsuji et al. 1996). A recent meta-analysis showed that this relationship is stronger in persons >45 years of age compared to <45 years of age (Panaite et al. 2015). Furthermore, they showed that HRR was most robust in predicting all-cause mortality. In patients with heart failure, HRR after maximal exercise remains a significant predictor irrespective of beta blockers (Arena et al. 2010). HRR after submaximal exercise also seems to have prognostic power in heart failure patients (Cahalin et al. 2013).

The prognostic importance of HRR is evident but the origin of the individual differences in HRR and vagal rebound after exercise remain unclear. The two main factors creating individual differences are innate biological differences and environmental factors. The latter can be subdivided in influences shared with other family members (common environmental influences; e.g. upbringing) and unique environmental influences (e.g. physical activity at work). Twin studies enable us to decompose the total variance in traits, e.g. HRR, into genetic, common environmental, and unique environmental components. Intrapair resemblance in HRR is compared between two types of twins; genetically identical (monozygotic, MZ) and non-identical (dizygotic, DZ) twins. When the MZ intrapair resemblance for HRR is higher than the DZ intrapair resemblance, this constitutes evidence for genetic influences on HRR. When the MZ resemblance for HRR is comparable to the DZ resemblance, this constitutes evidence for common environmental influences on HRR. The degree to which MZ twins are discordant for HRR indicates the influence of unique environmental influences on HRR. Structural equation modeling of the MZ and DZ twin covariances allows an estimation of the relative contribution of genetic influences on HRR to its total variance, an estimate known as the heritability of HRR.

Regular exercise behavior has been cited as a potential causal source of differences in resting heart rate and cardiac vagal control and is, therefore, also expected to influence HRR and vagal rebound (Billman 2002; Bosquet et al. 2007; Buch et al. 2002). Because regular exercise behavior has been shown to be a heritable trait (de Geus et al. 2014) it could be an important mediator of genetic effects on HRR and vagal rebound. Such hypotheses can be tested in twin models using a multivariate extension of the MZ and DZ twin (co)variance analysis. Work of Kupper et al. (Kupper et al. 2005), for instance, has already shown that the correlation between resting heart rate and resting cardiac vagal control could for a large extent be explained by genetic factors. Moreover, a large amount (>80 %) of the phenotypic correlation between exercise behavior and resting heart rate and between exercise behavior and resting cardiac vagal control could indeed be explained by genetic factors (de Geus et al. 2003). Whether a similar genetic contribution is seen when exploring the association between regular exercise behavior and HRR and vagal rebound after exercise remains unknown.

The first aim of this study is to estimate the heritability of immediate HRR (60 s after exercise cessation) and also of HRR at 3 min after exercise and the degree of vagal rebound measured by heart rate variability in the respiratory frequency range (RSA) at these time periods. Multivariate genetic modeling was used to test two further hypotheses: (1) the genetic factors influencing post-exercise HRR and vagal rebound do not completely overlap with those influencing resting heart rate and vagal control; (2) heritability of this ‘cardiac vagal exercise recovery’ factor in part reflects the heritability of regular voluntary exercise behavior. The latter hypothesis is optimally tested in a healthy adolescent population where exercise behavior is known to be highly heritable (72–80 %) (van der Aa et al. 2010).

## Methods

### Participants

Twin pairs aged between 16 and 18, enrolled in longitudinal survey studies of the Netherlands Twin Register (van Beijsterveldt et al. 2013) were invited to participate. Siblings within an age range of 12–25 years were also invited. Participants were excluded when having a history of cardiovascular or respiratory disease, or when being physically incapable of engaging in exercise activities. Eventually, 491 healthy adolescents participated in the study including 225 complete twin pairs: 58 monozygotic male pairs, 36 dizygotic male pairs, 56 monozygotic female pairs, 42 dizygotic female pairs, 33 dizygotic opposite sex pairs and 37 of their singleton siblings (56 % female). Mean age at time of their visit was 17.1 ± 1.1 years (range 12–25). All participants and, in participants under 18, both (one in the case of single parent families) of their parents/guardians provided written informed consent. All study procedures were reviewed and approved by the Medical Ethics Review Committee of the VU University Medical Center Amsterdam (NL35634.029.10).

### Electrocardiogram registration

Electrocardiogram (ECG) registration was done using VU-AMS device (Vrije Universiteit Ambulatory Monitoring System) (de Geus et al. 1995). R-peaks were scored by an algorithm within the VU-AMS software package and, if present, Premature Ventricular Contractions (PVCs) were scored by a trained researcher under close supervision of a cardiologist. All further analyses were done using PVC-free signals. Heart rate was calculated as an average of a five second period. An overview of calculation of heart rate measures used is displayed in Table 1.

**Table 1:** Description of variables

Variable	Description
Resting heart rate	Heart rate in number of beats per minute while sitting quietly
HRR60	Immediate heart rate recovery Calculated as: maximal heart rate—heart rate at 1 min after exercise
HRR180	Long-term heart rate recovery Calculated as: maximal heart rate—heart rate at 3 min after exercise
Resting RSA	Resting respiratory sinus arrhythmia Calculated via peak valley method while sitting quietly by averaging RSA in all breaths during 6 min of quiet sitting
RSA60	Immediate vagal rebound Respiratory sinus arrhythmia, an average of the RSA across all breaths in the first minute after exercise
RSA300	Long-term vagal rebound Respiratory sinus arrhythmia, an average of the RSA across all breaths in minute 2–5 after exercise

### Thoracic impedance and heart rate variability

In order to obtain breathing frequency, thoracic impedance was measured also using the VU-AMS device. Vagal rebound was assessed by HRV in the respiratory frequency range, extracted from the ECG and the respiratory signal (de Geus et al. 1995; Goedhart et al. 2007; Houtveen et al. 2006). Respiratory sinus arrhythmia (RSA) is defined as the longest heart period during expiration minus the shortest heart period during inspiration. RSA was computed on a breath-to-breath basis. Therefore, it is more robust than spectral decomposition methods against violation of the assumptions of stationarity. During exercise recovery such stationarity is likely low. When no difference in shortest and longest beats could be detected, RSA was set to be zero for that breath. An overview of calculation of RSA measures from the breath-to-breath data is displayed in Table 1.

### Exercise tests

Maximal exercise protocol was performed on a bike ergometer (Lode, type Corival) and consisted of an increasing workload per minute. Participants were encouraged by a researcher to exercise until exhaustion. Different recovery modes are in use (Barak et al. 2011). Active or supine recovery is preferred since it facilitates venous return and to this end reduces the risk of arrhythmias, hypotension and syncope post-exercise (Carter, III et al. 1999; Johnson et al. 1990; Takahashi et al. 2000). Also, since the participants were wearing equipment for the measurement of oxygen uptake on their back, supine recovery was unpractical. For these reasons, participants were obliged to remain seated on the bike ergometer for at least 5 min after cessation of the test for cool down. This cooldown was standardized as follows: after cessation of the test, the resistance was immediately decreased to 50 W (girls) or 70 W (boys) and participants were instructed to pedal at a comfortable rate between 30 and 60 rounds per minute. Resistance at recovery could be slightly adjusted on individual demand based on the maximally reached wattage during the test.

### Voluntary exercise behavior

Participants were queried on their voluntary exercise behavior (EB) as by the use of a short lifestyle interview described in detail elsewhere (van der Aa et al. 2010). The questions in this interview were structured similar to the longitudinal surveys in the Netherlands Twin Register. For each exercise activity (e.g. swimming, fitness, tennis, jogging, soccer) they were asked for how many years they have been doing that particular sport, how many months per year, how many times per week, and how many minutes each time. Participants had to have been active for at least 6 months and do it more than 3 months per year for the activity to be included as we were interested in regular exercise behavior. Hence, ski holidays, sailing camps, and similar were excluded. Also, activity related to transportation (cycling, walking) were excluded. Compulsory physical education classes were also excluded as they do not reflect voluntary exercise behavior and exercise activities are poorly standardized across different high schools in the Netherlands. Each remaining exercise activity was converted into a MET (metabolic equivalent task) score (Ridley et al. 2008). One MET represents the amount of energy needed for sitting quietly. This MET score was multiplied by the duration of activities and summed to get a weekly MET score. For instance, an individual reporting 90 min of field hockey training (MET score = 8) and a 70 min game each week would yield (2.67 h*8=) 21.3 METhours/weekly.

### Descriptive statistics

A multivariate Saturated model in OpenMx (Boker et al. 2011) under R (R Core Team 2014) was used to estimate the phenotypic correlations and their 95 % confidence intervals between EB, resting heart rate, resting RSA, HRR60, HRR180, RSA60 and RSA300 taking into account the nested structure of family data. To gain insight into the expected sources of variance in these measures and the underlying sources of phenotypic correlation between these measures MZ twin correlations and DZ/sibling correlations and cross-twin cross trait correlations were estimated.

### Genetic modeling

Genetic structural equation modeling was done with the raw-data ML procedure for estimation of parameters. For all analyses, a threshold of *p* < 0.05 was considered for statistical significance.

Because (non-twin) siblings share, like DZ twins, on average, 50 % of their segregating genes and since the sample size was rather small, parameter estimates were constrained to be equal for males and females and for DZ twins and siblings. However, main effects of sex and age on mean levels of the phenotypes were considered in the model.

**Fig. 1:**
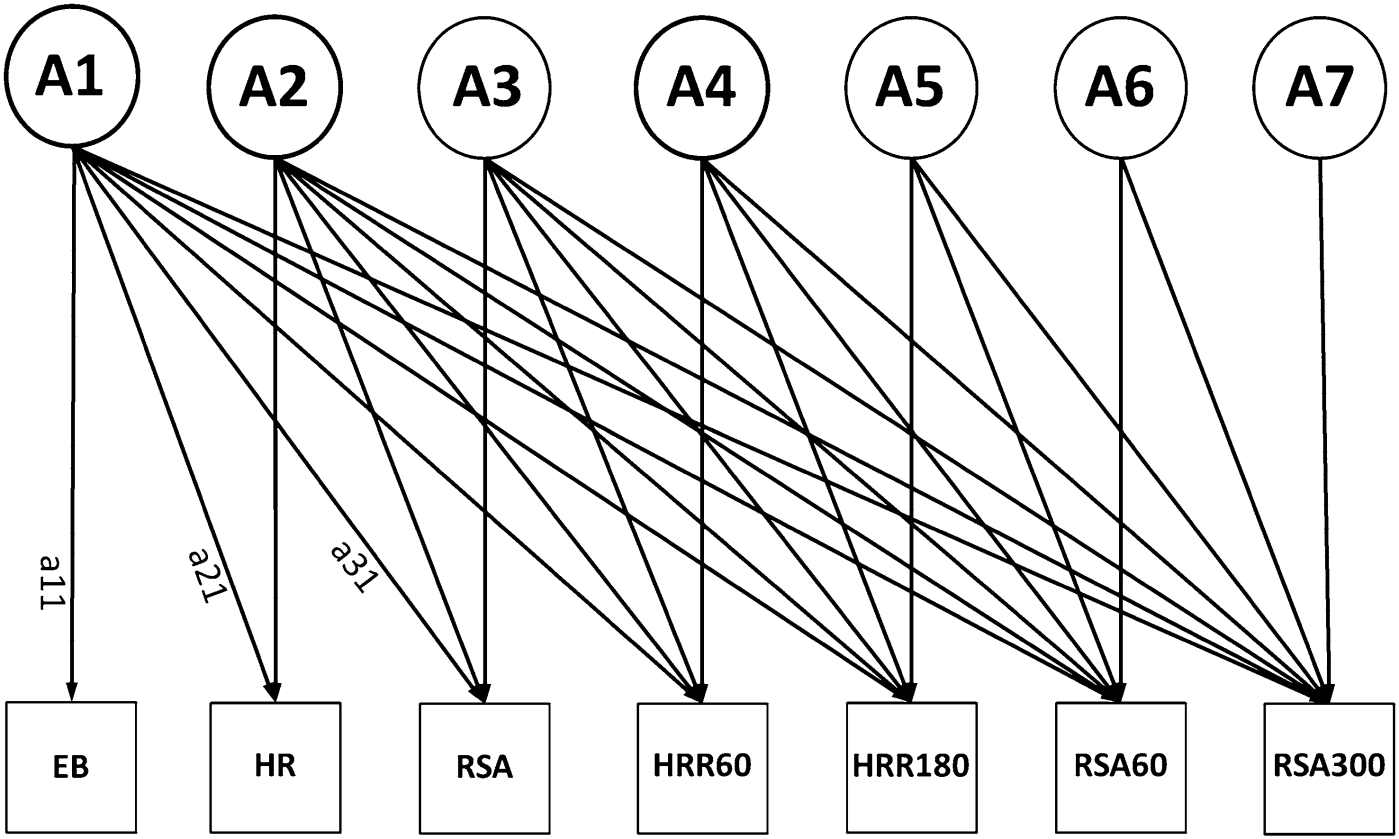
Full Seven variate Cholesky model (E not depicted in this figure). *HR*; resting heart rate. The path from factor *A1* to *EB* is named *a11*, the path from *A1* to HR *a21*; other path names follow the same reasoning

In multivariate models, the total phenotypic variance was decomposed into sources of additive genetic (co)variance (A), dominant genetic (co)variance (D) or common environmental (co)variance (C) and unique environmental (co)variance (E). Since C and D effects cannot be estimated simultaneously in the classical twin model, the ratio of the MZ correlations to the DZ correlations was used to determine which model (ACE or ADE) is most appropriate. Significance of variance components was tested by comparing the model including the specific component to a model in which the component is constraint to be equal to zero. This nested submodel was compared using hierarchic Chi-square tests. The Chi-square statistic is computed by subtracting log-likelihood (−2LL) for a reduced model from the −2LL for the full model (*χ* ^*2*^ = −2LL_full model_ – (−2LL_reduced model_)). This *χ* ^*2*^statistic is distributed with degrees of freedom (*df*) equal to the difference in the number of parameters estimated in the two models (Δ*df* = *df*_full model_ − *df*_reduced model_). If the difference test is significant the constraints on the reduced model cause a significant deterioration of the fit of the model and thus the component will be retained. In a multivariate saturated model (in which all parameters are estimated freely), we started the genetic model fitting with a Cholesky decomposition (Neale and Cardon 1992) for these seven variables (model 1). This Cholesky decomposition reveals a first insight into the etiology of covariances between variables. The model is descriptive and not driven by a specific multivariate hypothesis. Next, we tested for the significance of shared environmental influences by constraining these effects to be zero (model 2). This AE model is depicted in Fig. 1.

To test our hypotheses, the significance of selected path loadings was tested by constraining them to zero. This was done in three steps: first, we tested whether all factors influencing resting heart rate and resting vagal tone (see Fig. 1; A2 and A3) also influenced HRR and vagal rebound after exercise. All path loadings with zero in their 95 % confidence interval originating from genetic factor A2 and A3 (pathway *a22*, *a32*, *a42*, *a52*, *a62*, *a72*, *a33*, *a43*, *a53*, *a63 and a73* in Fig. 1) were constrained to be zero and the fit of this reduced model (model 3) was compared to the Cholesky AE model (model 2). Next, to test the hypothesis that a single genetic factor influences post-exercise HRR and vagal rebound overlap, path loadings of A4 through A7 (pathway *a44*, *a54*, *a64*, *a74*, *a55*, *a65*, *a75*, *a66*, *a76 and a77* in Fig. 1) with zero in their 95 % confidence interval were constrained to zero and this reduced model (model 4) was compared with model 3. Finally, the overlap between the genetic factor influencing regular exercise behavior and the cardiac vagal exercise recovery factors was explored by constraining the non-significant path loadings originating from A1 (pathway *a11*, *a21*, *a31*, *a41*, *a51*, *a61 and a71* in Fig. 1) to zero. The fit of this model (model 5) was then compared to model 4. In the final model only the significant pathways were retained and all heritability’s were computed using this final model.

## Results

### General descriptives

Means and standard deviations for resting heart rate, resting RSA, immediate HRR and vagal rebound, long-term HRR and vagal rebound, EB and ventricular arrhythmia (VA) are shown in Table 2. For the analysis of heart rate and HRV, further analyses were done using PVC-free signals; subjects who had ectopic beats were not excluded from the analysis but ectopic beats were removed from the data. 381 (77 %) adolescents showed no ventricular ectopy during the entire exercise protocol. 94 (19 %) had at least one but less than 10 premature ventricular contractions (PVCs) and 16 (3 %) had more than 10 PVCs during the entire recording. There was no difference between males and females (*p* = 0.178). Within the group of adolescents with PVCs, these were polymorph in 10 (9 %), bigeminal in 6 (5.5 %) and three adolescents showed one or more couplets (2.7 %). In the 16 adolescents whose ECG showed over 10 PVCs during the entire protocol, 13 showed one or more in rest, 10 during submaximal exercise and 6 during the maximal exercise test (see Table 2). Athletes (defined as >50 MET EB per week, *N* = 46) showed significantly more ventricular ectopy compared to non-athletes (*p* < 0.001). Variance in the presence of VA in this group could not be explained by genetic influences (data not shown).

**Table 2:** Descriptives

	Males		Females	
	Mean	SD	Mean	SD
Age	17.0	1.1	17.2	1.1
Ventricular arrhythmia (#PVCs)	1.6	8.0	2.7	23.5
None (*N*, (%))	183 (76)		198 (79)	
<10 (*N*, (%))	50 (21)		44 (18)	
>10 (*N*, (%))	7 (3)		9 (4)	
Resting heart rate (bpm)*	72.4	11.3	75.7	11.1
Maximal heart rate (bpm)	195.5	9.7	194.4	8.9
HRR60 (bpm)*	25.4	7.8	22.1	7.2
HRR180 (bpm)*	58.6	10.5	53.4	10.5
Resting RSA (ms)	74.9	38.0	80.2	41.1
RSA60 (ms)*	8.2	2.1	7.0	1.9
RSA300 (ms)*	9.7	4.1	8.4	3.9
EB (METs/week) *	26.0	22.6	19.3	21.7

*PVC* premature ventricular contraction

* Significant difference between males and females

### Heritability

**Fig. 2:**
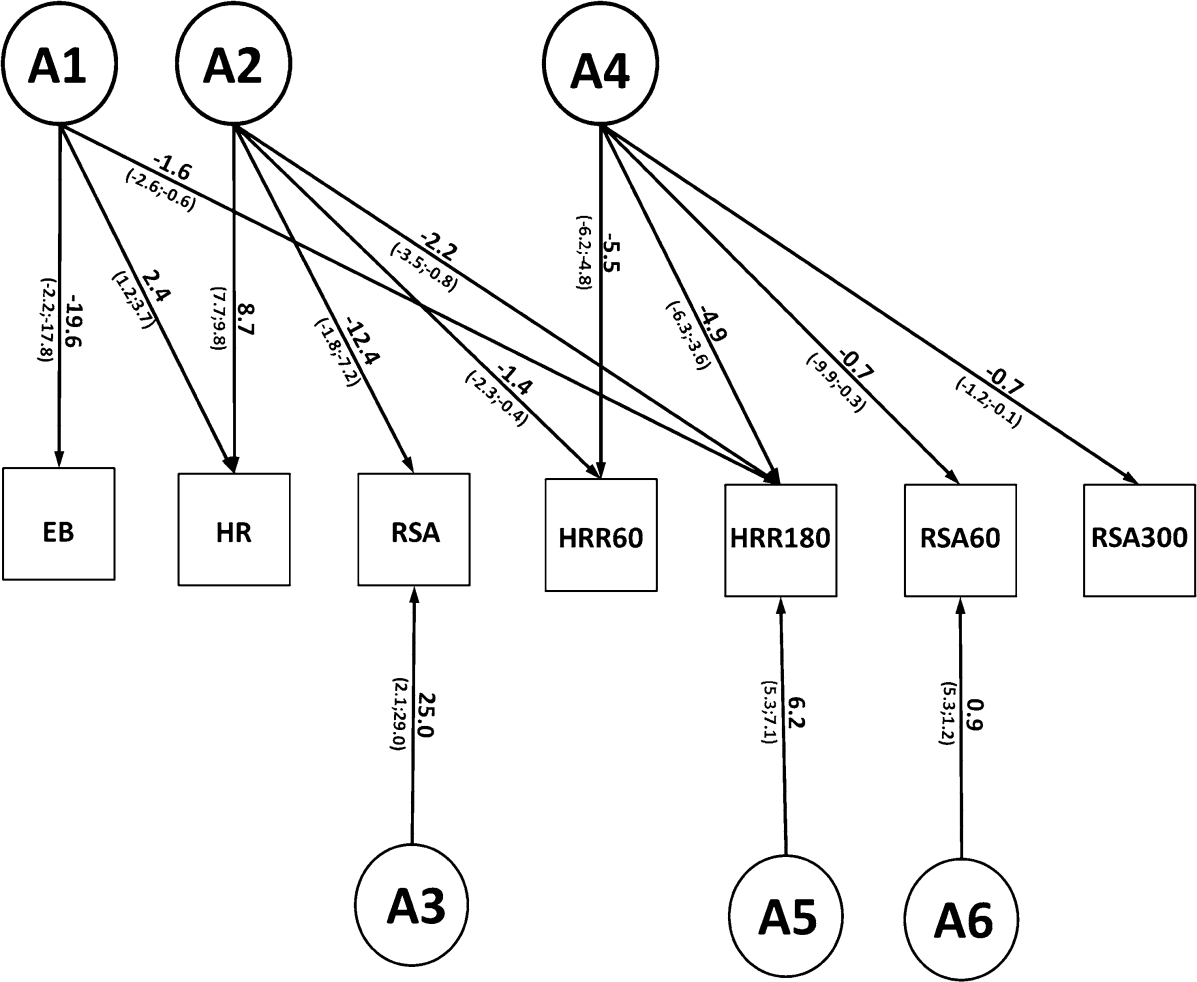
Reduced Cholesky model (E not depicted in this figure). *HR*; resting heart rate. Factor *A1*, *A2* and *A4* represent genetic influences which are partly shared. Tentatively, *A1* can be labeled as an exercise/heart rate factor, *A2* as a resting vagal tone and *A4* as the cardiac vagal recovery factor. The numbers next to the *arrows* represent the unstandardized path coefficients and their 95 % confidence intervals. Heritability can be computed by dividing the summed genetic variance by the total variance

Correlations for MZ twins were higher than DZ/sibling correlations for all phenotypes (see Table 3), suggesting a genetic effect. The results of the model fitting can be found in the Table 5. Based on the best fitting model (Fig. 2) we estimated a heritability of 80 % (95 % CI 73–85) for EB, 68 % (95 % CI 58–76) for resting heart rate, 60 % (95 % CI 48–67) for HRR60, 65 % (95 % CI 54–73) for HRR180, 58 % (95 % CI 45–69) for resting RSA, 23 % (95 % CI 11–35) for RSA60 and 3 % (95 % CI 0–11; not significant) for RSA300 (see Table 3). The remaining variance in these variables could be explained by unique environmental factors.

**Table 3:** MZ twin and DZ/sibling correlations were estimated in the multivariate saturated model and proportion of variance and confidence intervals due to A and E, were estimated from best fitting multivariate model

	*r*_MZ_	*r*_DZ/sib_	*A*	*E*
Resting heart rate	0.70 (0.60–0.78)	0.49 (0.36–0.59)	0.68 (0.58–0.76)	0.32 (0.24–0.42)
HRR60	0.61 (0.49–0.70)	0.38 (0.23–0.51)	0.60 (0.48–0.67)	0.40 (0.31–052)
HRR180	0.67 (0.56–0.75)	0.40 (0.27–0.51)	0.65 (0.54–0.73)	0.35 (0.27–0.46)
Resting RSA	0.64 (0.53–0.73)	0.19 (0.04–0.32)	0.58 (0.45–0.69)	0.42 (0.31–0.55)
RSA60	0.35 (0.19–0.49)	0.22 (0.06–0.37)	0.23 (0.11–0.35)	0.77 (0.65–0.89)
RSA300	0.24 (0.08–0.40)	0.15 (–0.04–0.33)	0.03 (0.00–0.11)	0.97 (0.89–1.00)
EB	0.80 (0.73–0.85)	0.48 (0.35–0.59)	0.80 (0.73–0.85)	0.20 (0.15–0.27)

### Genetic contributions to covariances

Phenotypic correlations are shown in Table 4. Resting heart rate was moderately correlated to resting RSA (*r* = −0.39, 95 % CI −0.47, −0.30) and showed a small inverse correlation to HRR and vagal rebound (−0.17 < *r* < −0.29).

**Table 4:**
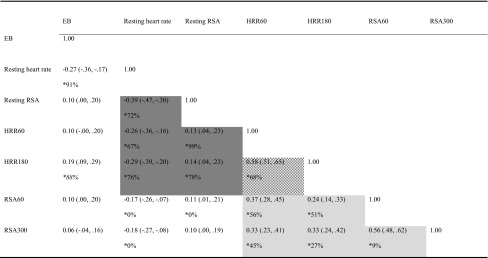
Phenotypic correlations (95 %CI) and in significant correlations % explained by overlapping genetic factors (*) estimated in multivariate models

HRR was moderately correlated to vagal rebound, even when short- and long-term recovery was cross-correlated (0.24 < *r* < 0.58). EB was significantly correlated to resting heart rate and HRR180 only. Table 4, therefore, suggest the existence of a general cardiac vagal factor that influences resting heart rate and resting RSA but also HRR and vagal rebound; dark gray shaded area) and a specific cardiac vagal exercise recovery factor (including HRR60, HRR180, RSA60 and RSA300; light gray shaded area).

The existence of these factors were confirmed by the Cholesky model. Pathloadings *a62*, *a72*, *a43*, *a53*, *a63*, *a43*, *a53*, *a63* and *a73*, *a65*, *a75*, *a76*, *a77*, *a31*, *a41*, *a61* and *a71* were constrained to zero as zero was in the 95 % confidence interval. The final model is shown in Fig. 2. Factor A2 loaded on resting heart rate and resting vagal tone, as well as HRR60 and HRR180 and might be considered as a general cardiac vagal factor. The majority of the phenotypic correlations between these variables could be explained by genetic factors (72–99 %). Factor A4 can be considered as a recovery factor, as significant path loadings were detected between this factor and all four recovery variables. 9–68 % of the phenotypic correlations between these recovery variables could be explained by genetic factors. Finally, the same genetic factor (A1) that influenced regular exercise behavior also influenced resting heart rate and long-term heart rate recovery. Almost all (88–91 %) of the observable phenotypic correlation between these variables could be explained by this common genetic factor. Model fitting results can be found in Table 5.

**Table 5:** Model fitting results

Model	−2LL	*df*	Compared to	*Χ* ^2^	Δ*df*	*p*
1 Cholesky ACE	23,466	3257				
2 Cholesky AE	23,488	3285	1	22	28	0.816
3 Cholesky AE—HR and resting vagal tone	23,500	3291	2	12	6	0.063
4 Cholesky AE—HRR and vagal rebound	23,506	3295	3	6	4	0.193
5 Cholesky AE—vagal exercise recovery and EB	23,512	3299	4	6	4	0.186

## Discussion

Heart rate responses to (maximal) exercise exhibit important prognostic power for cardiovascular and total mortality. Immediate HRR (1 min after exercise cessation) is an easy to measure and potentially very valuable predictor and a measure of cardiac vagal function in patients as well as in healthy individuals. This study in a group of healthy adolescent twins and their siblings estimated the heritability of HRR and vagal rebound after maximal exercise. In this group, estimated heritability of immediate HRR60 was 60 % (95 % CI 48–67). The immediate vagal rebound, measured by RSA60 in this study, showed a heritability estimate of 23 % (95 % CI 11–35). The long-term vagal rebound showed heritability estimates of 65 % (95 % CI 54–73) for HRR180 but only 3 % (95 % CI 0–11) for RSA300. Another limitation of this study is that maturational age may influence both heart rate and HRV and maturation is likely to be more synchronous in MZ than in DZ twins which may have inflated heritability. In contrast, the broad age range of the siblings could have acted to inflate the environmental effects.

Previous studies had already firmly established the heritability of resting heart rate with estimates varying from 26 to 68 % (An et al. 1999; de Geus et al. 2007; Singh et al. 1999; Wang et al. 2015; Zhang et al. 2014). Likewise resting RSA is substantially heritable with estimates ranging between 25 and 71 % (de Geus et al. 2015; Neijts et al. 2014). We replicate these findings in our sample (heart rate 68 %, RSA 58 %) with the heritability of resting heart rate very similar to a recent meta-analysis done by Wang et al. (Wang et al. 2015) and the heritability of resting RSA very similar to that found in a large non-overlapping study in adult Dutch twins (50 %) (Neijts et al. 2015). To our knowledge, only one previous study has been done on the heritability of HRR and none on other vagal rebound measures. Ingelsson et al. (Ingelsson et al. 2007) found a heritability of 34 % for slow HRR (3 min after cessation of exercise), which is much lower compared to our results (65 %). This is probably due to the different methods used; participants did not exercise until exhaustion as in our study but until they reached 85 % of their estimated maximal heart rate. Also, recovery was in supine position, whereas our participants had an active recovery.

Previously, Kupper et al. (Kupper et al. 2005) showed that the phenotypic correlations between heart rate and RSA measured in an ambulatory setting (ranging between 0.35 and 0.45, measured at different times of day) was for up to 52 % determined by common genetic factors. We here replicate this finding for resting levels of heart rate and RSA in a more standardized setting. In our study, the phenotypic correlation between resting heart rate and resting RSA was −0.39, and 72 % of this correlation could be explained by a common genetic factor influencing both resting heart rate and resting RSA. We now furthermore, show that the general cardiac vagal factor influencing resting heart rate and RSA also influences HRR and vagal rebound to exercise. This could not simply have been assumed a priori. Dewland et al. have suggested that different physiologic determinants probably underlie resting heart rate and HRR (Dewland et al. 2007) and this was further reinforced by the findings of Duarte and colleagues (Duarte et al. 2015). Participants to their training study were divided based on their resting vagal control being high or low. Post-exercise vagal rebound showed no change in a control group, but training 3 days per week for 40 min at 75–85 % of their heart rate reserve led to a significant increase in maximal oxygen consumption and post-exercise vagal rebound. Resting vagal control, however, only increased for the group with low resting vagal control at baseline but not in the group with high resting vagal control at baseline. Phenotypic correlations between the heart rate and RSA variables in our study were in keeping with the existence of two different factors: a ‘general cardiac vagal factor’ (including resting heart rate, resting RSA and the vagal rebound effects after exercise; dark gray shaded area in Table 4) and a more specific ‘cardiac vagal exercise recovery’ factor (including immediate HRR and vagal rebound and long-term HRR and vagal rebound; light gray shaded area in Table 4).

Voluntary exercise behavior is a highly heritable trait, 80 % in our study corresponds well to an earlier study in an unrelated adolescent cohort (van der Aa et al. 2010). Heritability of EB increases from childhood to adolescence (Huppertz et al. 2016) meaning that the likelihood of success of family based interventions is largest in children and less in adolescents. For adolescents, focus should shift to individually based interventions.

Regular exercise behavior and HRR have been reported to be significantly associated in patients (Rodrigues et al. 2015; Sato et al. 2005) and in healthy persons (Carnethon et al. 2005). In our study, the correlations between regular exercise and HRR60, HRR180, RSA60 and RSA300 were low (0.06–0.19) and only HRR180 reached significance (*r* = 0.19). Albeit small, the correlation between EB and HRR180 was almost entirely explained by genetic factors (88 %). Such a finding can be ascribed to (a combination of) three possible explanations. First, regular exercise behavior may causally influence long-term cardiac vagal recovery, in which case the genes for exercise behavior are part of the heritability of cardiac vagal recovery. In this scenario, changing exercise behavior would positively influence this phenotype. Second, the reverse may also be true in that long-term cardiac vagal recovery may causally influence regular exercise behavior. Third, there may be genetic pleiotropic effects that independently influence exercise behavior and vagal recovery. The latter two explanations are less likely, as in that case intervention on exercise behavior would not be expected to change vagal recovery. EB also correlated significantly with resting heart rate (*r* = −0.27, 95 % CI −0.36, −0.17), of which 91 % could be explained by genetic factors. The well-known phenomenon of bradycardia in athletes is mostly due to lowered intrinsic heart rate, although vagal control also plays a minor role (Katona et al. 1982; Smith et al. 1989). This is in accordance with our current results, as EB shows a significant correlation with resting heart rate and HRR180 but not with the other variables. A possible explanation for the bradycardia is increased stretch of the right atrium and thereby the sino-atrial and the atrio-ventricular node induced by the chronically increased stroke volume.

In conclusion, individual differences in HRR can to a large extent be explained by genetic differences. Because our results reveal partly different genetic determinants for general and post-exercise cardiac vagal control, we suggest that for heart rate and HRV, both resting and post-exercise values should be considered when evaluating differences in autonomic nervous system control as predictors of cardiac and all-cause mortality.

## Abbreviations

A: Additive genetic (co)variance
C: Common environmental (co)variance
CHRM2: Catecholamine receptor M2
D: Dominant genetic (co)variance
DZ: Dizygotic
E: Unique environmental (co)variance
EB: Exercise behavior
ECG: Electrocardiogram
HRR: Heart rate recovery
HRV: Heart rate variability
LL: Log-likelihood
MET: Metabolic equivalent task
MZ: Monozygotic
PVC: Premature ventricular contraction
RSA: Respiratory sinus arrhythmia
VA: Ventricular arrhythmia

## References

[CR1] An P, Rice T, Gagnon J, Borecki IB, Perusse L, Leon AS, Skinner JS, Wilmore JH, Bouchard C, Rao DC (1999). Familial aggregation of resting blood pressure and heart rate in a sedentary population: the HERITAGE family study. Health, risk factors, exercise training, and genetics. Am J Hypertens.

[CR2] Arena R, Myers J, Abella J, Peberdy MA, Bensimhon D, Chase P, Guazzi M (2010) The prognostic value of the heart rate response during exercise and recovery in patients with heart failure: influence of beta-blockade. Int J Cardiol 138(2):166–173 10.1016/j.ijcard.2008.08.010 18804882

[CR3] Barak OF, Ovcin ZB, Jakovljevic DG, Lozanov-Crvenkovic Z, Brodie DA, Grujic NG (2011). Heart rate recovery after submaximal exercise in four different recovery protocols in male athletes and non-athletes. J Sports Sci Med.

[CR4] Berntson GG, Cacioppo JT, Quigley KS (1993). Respiratory sinus arrhythmia: autonomic origins, physiological mechanisms, and psychophysiological implications. Psychophysiology.

[CR5] Bigger JT, Jr., Fleiss JL, Rolnitzky LM, Steinman RC (1993) Frequency domain measures of heart period variability to assess risk late after myocardial infarction. J Am Coll Cardiol 21(3):729–736 10.1016/0735-1097(93)90106-b 8436755

[CR6] Billman GE (2002) Aerobic exercise conditioning: a nonpharmacological antiarrhythmic intervention. J Appl Physiol (1985) 92(2):446–454 10.1152/japplphysiol.00874.2001 11796650

[CR7] Boker S, Neale M, Maes H, Wilde M, Spiegel M, Brick T, Spies J, Estabrook R, Kenny S, Bates T, Mehta P, Fox J (2011) OpenMx: an open source extended structural equation modeling framework. Psychometrika. 76(2):306–317 10.1007/s11336-010-9200-6 PMC3525063 23258944

[CR8] Bonilla IM, Belevych AE, Sridhar A, Nishijima Y, Ho HT, He Q, Kukielka M, Terentyev D, Terentyeva R, Liu B, Long VP, Gyorke S, Carnes CA, Billman GE (2012) Endurance exercise training normalizes repolarization and calcium-handling abnormalities, preventing ventricular fibrillation in a model of sudden cardiac death. J Appl Physiol (1985) 113(11):1772–1783 10.1152/japplphysiol.00175.2012 PMC3544509 23042911

[CR9] Bosquet L, Gamelin FX, Berthoin S (2007). Is aerobic endurance a determinant of cardiac autonomic regulation?. Eur J Appl Physiol.

[CR10] Buch AN, Coote JH, Townend JN (2002). Mortality, cardiac vagal control and physical training—what’s the link?. Exp Physiol.

[CR11] Cahalin LP, Arena R, Labate V, Bandera F, Lavie CJ, Guazzi M (2013). Heart rate recovery after the 6 min walk test rather than distance ambulated is a powerful prognostic indicator in heart failure with reduced and preserved ejection fraction: a comparison with cardiopulmonary exercise testing. Eur J Heart Fail.

[CR12] Carnethon MR, Jacobs DR, Sidney S, Sternfeld B, Gidding SS, Shoushtari C, Liu K (2005). A longitudinal study of physical activity and heart rate recovery: CARDIA, 1987–1993. Med Sci Sports Exerc.

[CR13] Carter R, III, Watenpaugh DE, Wasmund WL, Wasmund SL, Smith ML (1999) Muscle pump and central command during recovery from exercise in humans. J Appl Physiol (1985) 87(4):1463–1469 10.1152/jappl.1999.87.4.1463 10517779

[CR14] Cole CR, Foody JM, Blackstone EH, Lauer MS (2000) Heart rate recovery after submaximal exercise testing as a predictor of mortality in a cardiovascularly healthy cohort. Ann Intern Med 132(7):552–555 10.7326/0003-4819-132-7-200004040-00007 10744592

[CR15] Cole CR, Blackstone EH, Pashkow FJ, Snader CE, Lauer MS (1999) Heart-rate recovery immediately after exercise as a predictor of mortality. N Engl J Med 341(18):1351–1357 10.1056/NEJM199910283411804 10536127

[CR16] de Geus EJ, Willemsen GH, Klaver CH, Van Doornen LJ (1995) Ambulatory measurement of respiratory sinus arrhythmia and respiration rate. Biol Psychol 41(3):205–227 10.1016/0301-0511(95)05137-6 8608201

[CR17] de Geus EJ, Boomsma DI, Snieder H (2003). Genetic correlation of exercise with heart rate and respiratory sinus arrhythmia. Med Sci Sports Exerc.

[CR18] de Geus EJ, Kupper N, Boomsma DI, Snieder H (2007). Bivariate genetic modeling of cardiovascular stress reactivity: does stress uncover genetic variance?. Psychosom Med.

[CR19] de Geus EJ, Bartels M, Kaprio J, Lightfoot JT, Thomis M (2014). Genetics of regular exercise and sedentary behaviors. Twin Res Hum Genet.

[CR20] de Geus EJ, van Lien R, Neijts M, Willemsen (2015) Genetics of autonomic nervous system activity. The oxford handbook of molecular psychology. (17):357–390

[CR21] Dewland TA, Androne AS, Lee FA, Lampert RJ, Katz SD (2007). Effect of acetylcholinesterase inhibition with pyridostigmine on cardiac parasympathetic function in sedentary adults and trained athletes. Am J Physiol Heart Circ Physiol.

[CR22] Diller GP, Dimopoulos K, Okonko D, Uebing A, Broberg CS, Babu-Narayan S, Bayne S, Poole-Wilson PA, Sutton R, Francis DP, Gatzoulis MA (2006) Heart rate response during exercise predicts survival in adults with congenital heart disease. J Am Coll Cardiol 48(6):1250–1256 10.1016/j.jacc.2006.05.051 16979014

[CR23] Duarte A, Soares PP, Pescatello L, Farinatti P (2015). Aerobic training improves vagal reactivation regardless of resting vagal control. Med Sci Sports Exerc.

[CR24] Goedhart AD, van der Sluis S, Houtveen JH, Willemsen G, de Geus EJ (2007). Comparison of time and frequency domain measures of RSA in ambulatory recordings. Psychophysiology.

[CR25] Greenland P, Daviglus ML, Dyer AR, Liu K, Huang CF, Goldberger JJ, Stamler J (1999) Resting heart rate is a risk factor for cardiovascular and noncardiovascular mortality: the Chicago Heart Association Detection Project in Industry. Am J Epidemiol 149(9):853–862 10.1093/oxfordjournals.aje.a009901 10221322

[CR26] Groarke JD, Tanguturi VK, Hainer J, Klein J, Moslehi JJ, Ng A, Forman DE, Di Carli MF, Nohria A (2015) Abnormal exercise response in long-term survivors of hodgkin lymphoma treated with thoracic irradiation: evidence of cardiac autonomic dysfunction and impact on outcomes. J Am Coll Cardiol 65(6):573–583 10.1016/j.jacc.2014.11.035 25677317

[CR27] Heart rate variability (1996) Standards of measurement, physiological interpretation, and clinical use. Task Force of the European Society of Cardiology and the North American Society of Pacing and Electrophysiology. Eur Heart J 17(3):354–381 8737210

[CR28] Houtveen JH, Groot PF, de Geus EJ (2006). Validation of the thoracic impedance derived respiratory signal using multilevel analysis. Int J Psychophysiol.

[CR29] Huppertz C, Bartels M, de Zeeuw E, van Beijsterveldt CE, Hudziak JJ, Willemsen G, Boomsma DI, de Geus EJ (2016) Individual differences in exercise behavior: stability and change in genetic and environmental determinants from age 7–18. Behav Genet 10.1007/s10519-016-9799-x 27406597

[CR30] Imai K, Sato H, Hori M, Kusuoka H, Ozaki H, Yokoyama H, Takeda H, Inoue M, Kamada T (1994) Vagally mediated heart rate recovery after exercise is accelerated in athletes but blunted in patients with chronic heart failure. J Am Coll Cardiol 24(6):1529–1535 10.1016/0735-1097(94)90150-3 7930286

[CR31] Ingelsson E, Larson MG, Vasan RS, O’Donnell CJ, Yin X, Hirschhorn JN, Newton-Cheh C, Drake JA, Musone SL, Heard-Costa NL, Benjamin EJ, Levy D, Atwood LD, Wang TJ, Kathiresan S (2007) Heritability, linkage, and genetic associations of exercise treadmill test responses. Circ 115(23):2917–2924 10.1161/CIRCULATIONAHA.106.683821 17548724

[CR32] Johnson EC, Hudson TL, Greene ER (1990) Left ventricular hemodynamics during exercise recovery. J Appl Physiol (1985) 69(1):104–111 10.1152/jappl.1990.69.1.104 2394639

[CR33] Jouven X, Empana JP, Schwartz PJ, Desnos M, Courbon D, Ducimetiere P (2005) Heart-rate profile during exercise as a predictor of sudden death. N Engl J Med 352(19):1951–1958 10.1056/NEJMoa043012 15888695

[CR34] Katona PG, McLean M, Dighton DH, Guz A (1982) Sympathetic and parasympathetic cardiac control in athletes and nonathletes at rest. J Appl Physiol Respir Environ Exerc Physiol 52(6):1652–1657 10.1152/jappl.1982.52.6.1652 7107476

[CR35] Kleiger RE, Miller JP, Bigger JT, Jr., Moss AJ (1987) Decreased heart rate variability and its association with increased mortality after acute myocardial infarction. Am J Cardiol 59(4):256–262 10.1016/0002-9149(87)90795-8 3812275

[CR36] Kupper N, Willemsen G, Posthuma D, de Boer D, Boomsma DI, de Geus EJ (2005). A genetic analysis of ambulatory cardiorespiratory coupling. Psychophysiology.

[CR37] Lahiri MK, Kannankeril PJ, Goldberger JJ (2008) Assessment of autonomic function in cardiovascular disease: physiological basis and prognostic implications. J Am Coll Cardiol 51(18):1725–1733 10.1016/j.jacc.2008.01.038 18452777

[CR38] Lovallo WR (2015). Can exaggerated stress reactivity and prolonged recovery predict negative health outcomes? The case of cardiovascular disease. Psychosom Med.

[CR39] Minkkinen M, Nieminen T, Verrier RL, Leino J, Lehtimaki T, Viik J, Lehtinen R, Nikus K, Koobi T, Turjanmaa V, Kahonen M (2014) Prognostic capacity of a clinically indicated exercise test for cardiovascular mortality is enhanced by combined analysis of exercise capacity, heart rate recovery and T-wave alternans. Eur J Prev Cardiol 10.1177/2047487314557190 25366884

[CR40] Mora S, Redberg RF, Sharrett AR, Blumenthal RS (2005) Enhanced risk assessment in asymptomatic individuals with exercise testing and Framingham risk scores. Circ 112(11):1566–1572 10.1161/CIRCULATIONAHA.105.542993 16144993

[CR41] Neale M, Cardon L (1992) Methology for genetic studies of twins and families

[CR42] Neijts M, van Lien R, Kupper N, Boomsma D, Willemsen G, de Geus EJ (2014). Heritability of cardiac vagal control in 24-h heart rate variability recordings: influence of ceiling effects at low heart rates. Psychophysiology.

[CR43] Neijts M, van Lien R, Kupper N, Boomsma D, Willemsen G, de Geus EJ (2015). Heritability and temporal stability of ambulatory autonomic stress reactivity in unstructured 24-h recordings. Psychosom Med.

[CR44] Nissinen SI, Makikallio TH, Seppanen T, Tapanainen JM, Salo M, Tulppo MP, Huikuri HV (2003). Heart rate recovery after exercise as a predictor of mortality among survivors of acute myocardial infarction. Am J Cardiol.

[CR45] Ohuchi H, Suzuki H, Yasuda K, Arakaki Y, Echigo S, Kamiya T (2000). Heart rate recovery after exercise and cardiac autonomic nervous activity in children. Pediatr Res.

[CR46] Palatini P, Julius S (2004). Elevated heart rate: a major risk factor for cardiovascular disease. Clin Exp Hypertens.

[CR47] Panaite V, Salomon K, Jin A, Rottenberg J (2015). Cardiovascular recovery from psychological and physiological challenge and risk for adverse cardiovascular outcomes and all-cause mortality. Psychosom Med.

[CR48] Ridley K, Ainsworth BE, Olds TS (2008). Development of a compendium of energy expenditures for youth. Int J Behav Nutr Phys Act.

[CR49] Rodrigues P, Santos M, Sousa MJ, Brochado B, Anjo D, Barreira A, Preza-Fernandes J, Palma P, Viamonte S, Torres S (2015) Cardiac rehabilitation after an acute coronary syndrome: the impact in elderly patients. Cardiology 131(3):177–185 10.1159/000381824 25968103

[CR50] Sato S, Makita S, Majima M (2005). Additional physical activity during cardiac rehabilitation leads to an improved heart rate recovery in male patients after coronary artery bypass grafting. Circ J.

[CR51] Schomer AC, Nearing BD, Schachter SC, Verrier RL (2014). Vagus nerve stimulation reduces cardiac electrical instability assessed by quantitative T-wave alternans analysis in patients with drug-resistant focal epilepsy. Epilepsia.

[CR52] Schwartz AR, Gerin W, Davidson KW, Pickering TG, Brosschot JF, Thayer JF, Christenfeld N, Linden W (2003). Toward a causal model of cardiovascular responses to stress and the development of cardiovascular disease. Psychosom Med.

[CR53] Singh JP, Larson MG, O’Donnell CJ, Tsuji H, Evans JC, Levy D (1999) Heritability of heart rate variability: the Framingham Heart Study. Circ 99(17):2251–2254 10.1161/01.cir.99.17.2251 10226089

[CR54] Smith ML, Hudson DL, Graitzer HM, Raven PB (1989). Exercise training bradycardia: the role of autonomic balance. Med Sci Sports Exerc.

[CR55] Takahashi T, Okada A, Saitoh T, Hayano J, Miyamoto Y (2000). Difference in human cardiovascular response between upright and supine recovery from upright cycle exercise. Eur J Appl Physiol.

[CR56] Tsuji H, Larson MG, Venditti FJ, Jr., Manders ES, Evans JC, Feldman CL, Levy D (1996) Impact of reduced heart rate variability on risk for cardiac events. The Framingham Heart Study. Circ 94(11):2850–2855 10.1161/01.cir.94.11.2850 8941112

[CR57] van Beijsterveldt CE, Groen-Blokhuis M, Hottenga JJ, Franic S, Hudziak JJ, Lamb D, Huppertz C, de Zeeuw E, Nivard M, Schutte N, Swagerman S, Glasner T, van Fulpen M, Brouwer C, Stroet T, Nowotny D, Ehli EA, Davies GE, Scheet P, Orlebeke JF, Kan KJ, Smit D, Dolan CV, Middeldorp CM, de Geus EJ, Bartels M, Boomsma DI (2013). The Young Netherlands Twin Register (YNTR): longitudinal twin and family studies in over 70,000 children. Twin Res Hum Genet.

[CR58] van der Aa N, de Geus EJ, van Beijsterveldt TC, Boomsma DI, Bartels M (2010). Genetic influences on individual differences in exercise behavior during adolescence. Int J Pediatr.

[CR59] Vanoli E, De Ferrari GM, Stramba-Badiale M, Hull SS, Foreman RD, Schwartz PJ (1991). Vagal stimulation and prevention of sudden death in conscious dogs with a healed myocardial infarction. Circ Res.

[CR60] Wang B, Liao C, Zhou B, Cao W, Lv J, Yu C, Gao W, Li L (2015) Genetic contribution to the variance of blood pressure and heart rate: a systematic review and meta-regression of twin studies. Twin Res Hum Genet 1–13 10.1017/thg.2015.8 25744168

[CR61] Watanabe J, Thamilarasan M, Blackstone EH, Thomas JD, Lauer MS (2001) Heart rate recovery immediately after treadmill exercise and left ventricular systolic dysfunction as predictors of mortality: the case of stress echocardiography. Circ 104(16):1911–1916 11602493

[CR62] Zhang GQ, Zhang W (2009). Heart rate, lifespan, and mortality risk. Ageing Res Rev.

[CR63] Zhang K, Deacon DC, Rao F, Schork AJ, Fung MM, Waalen J, Schork NJ, Nievergelt CM, Chi NC, O’Connor DT (2014) Human heart rate: heritability of resting and stress values in twin pairs, and influence of genetic variation in the adrenergic pathway at a micro-ribonucleic acid (microrna) motif in the 3′-UTR of cytochrome b561 [corrected]. J Am Coll Cardiol 63(4):358–368 10.1016/j.jacc.2013.09.025 PMC3946708 24140660

